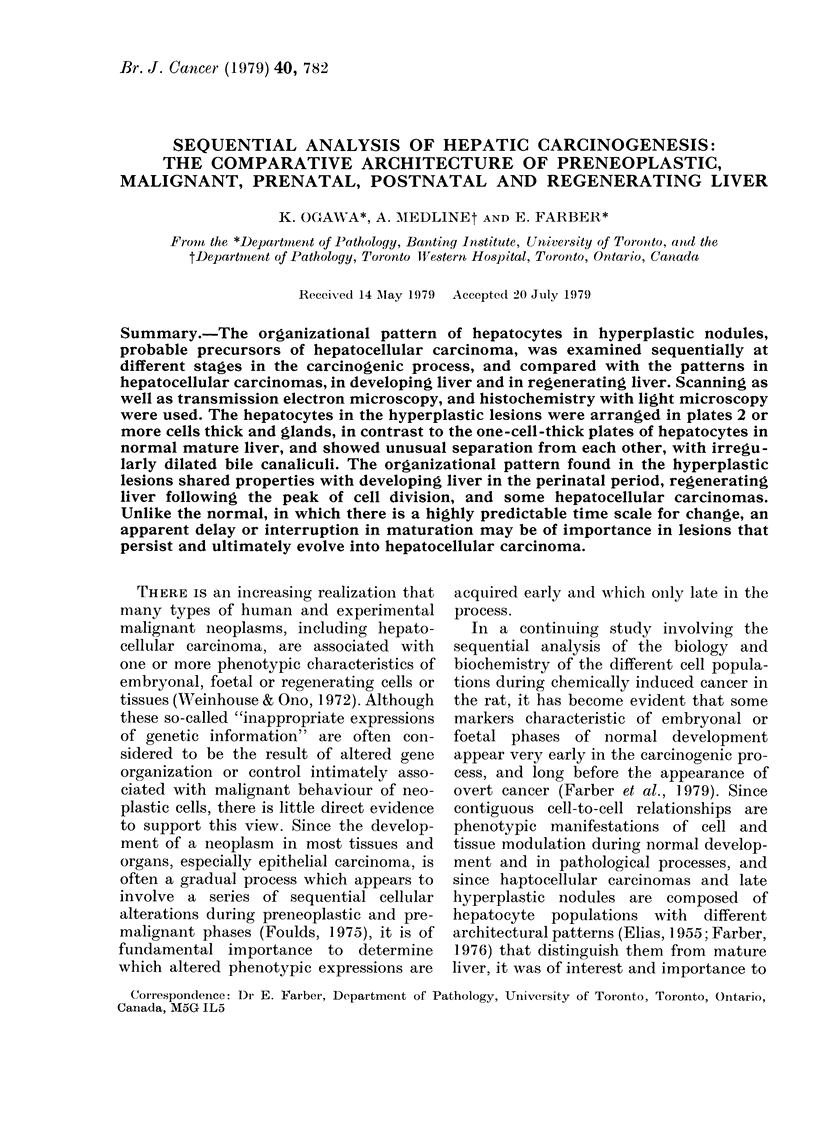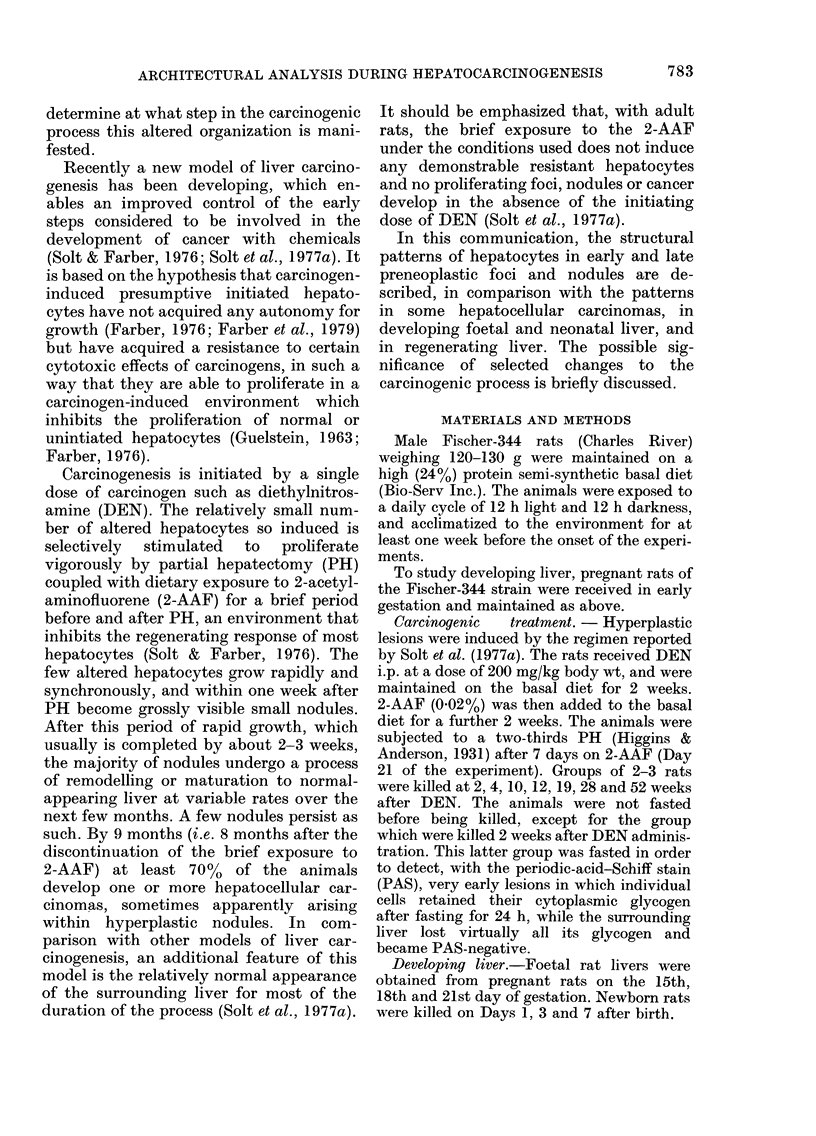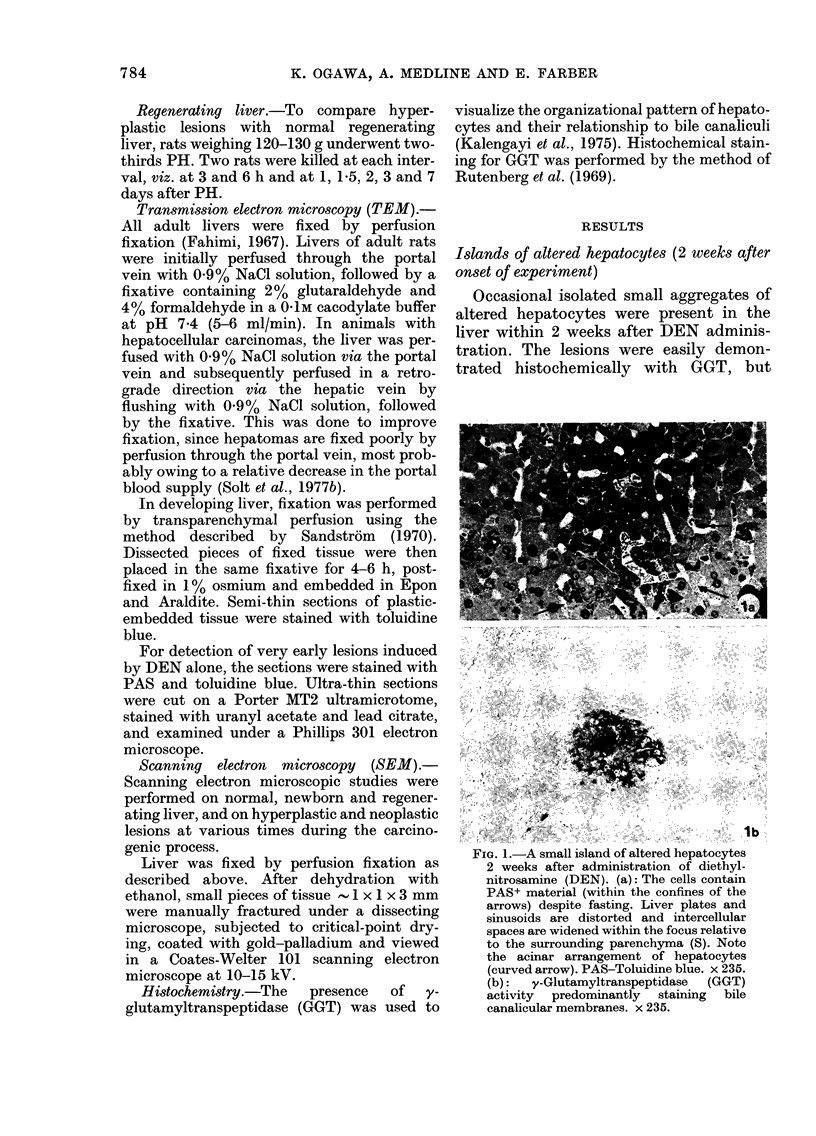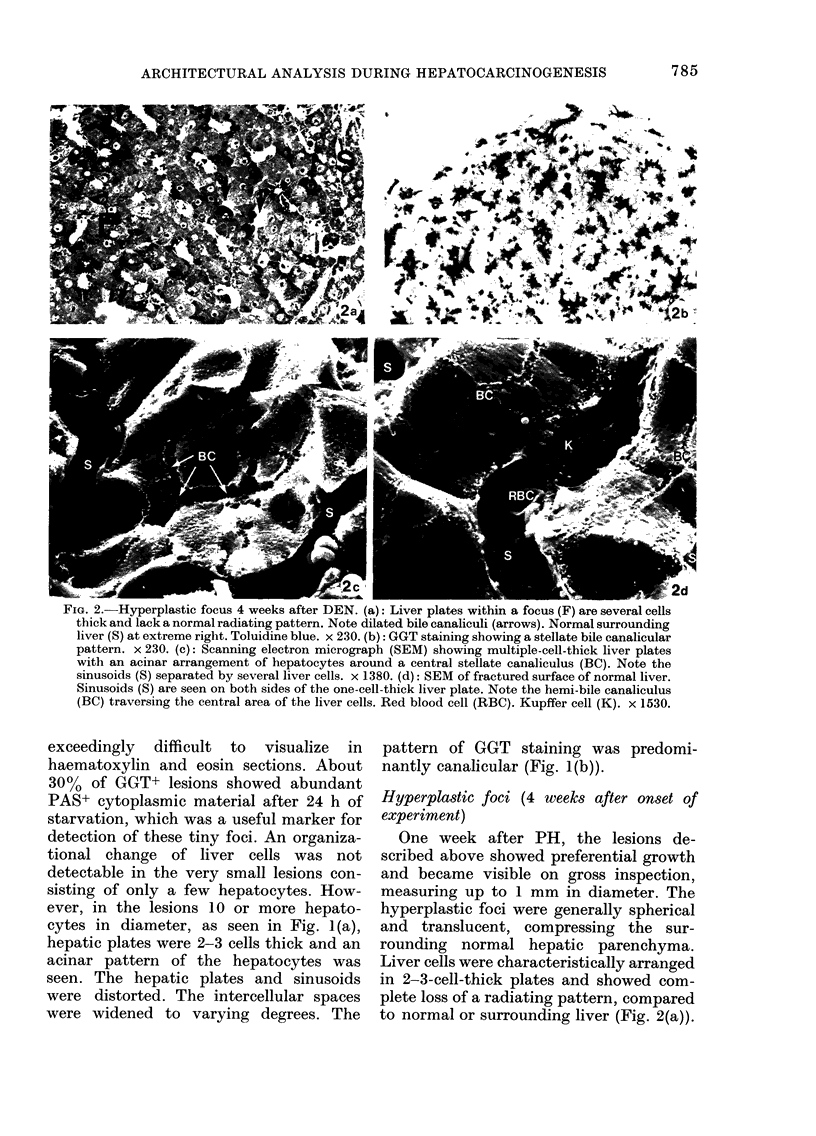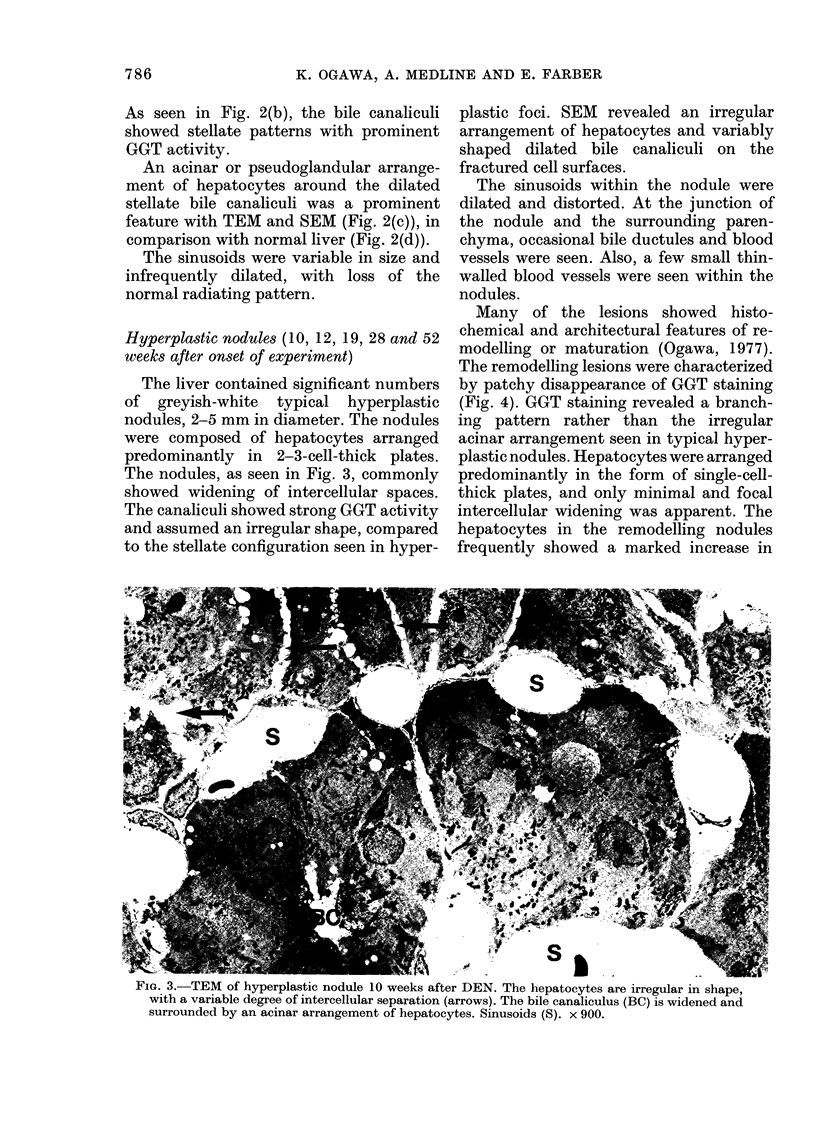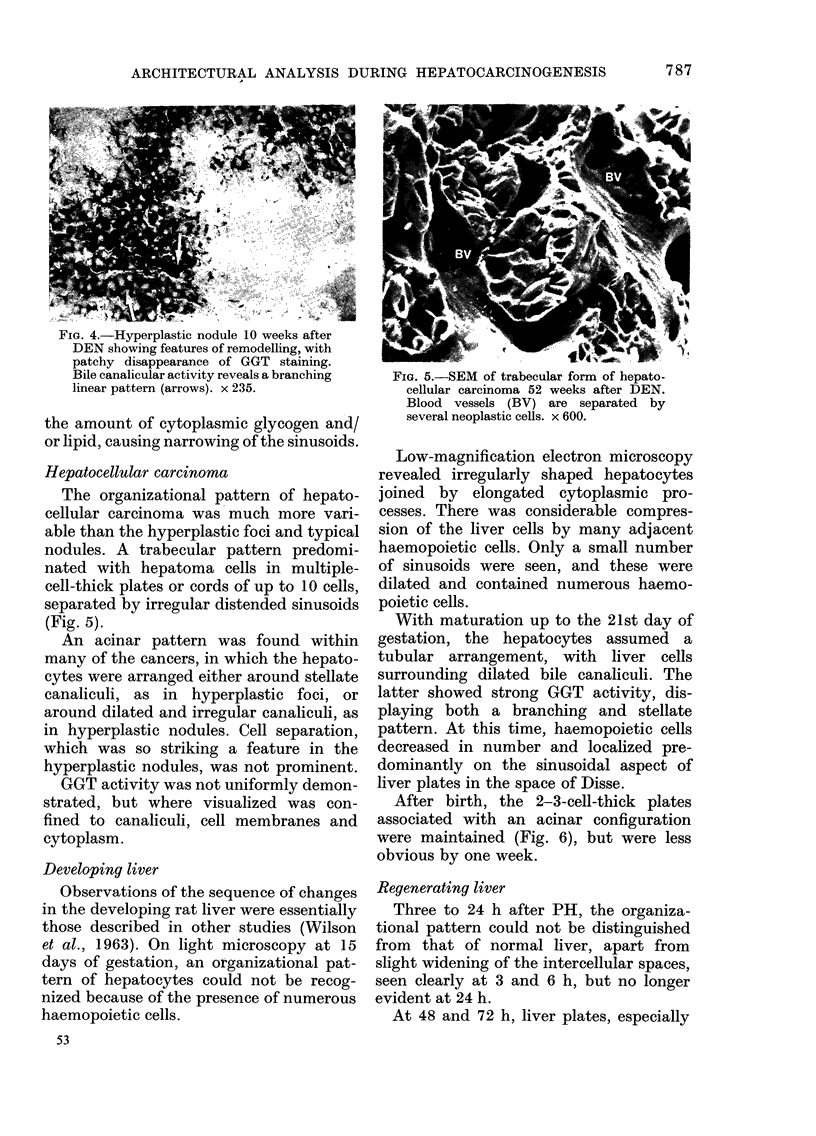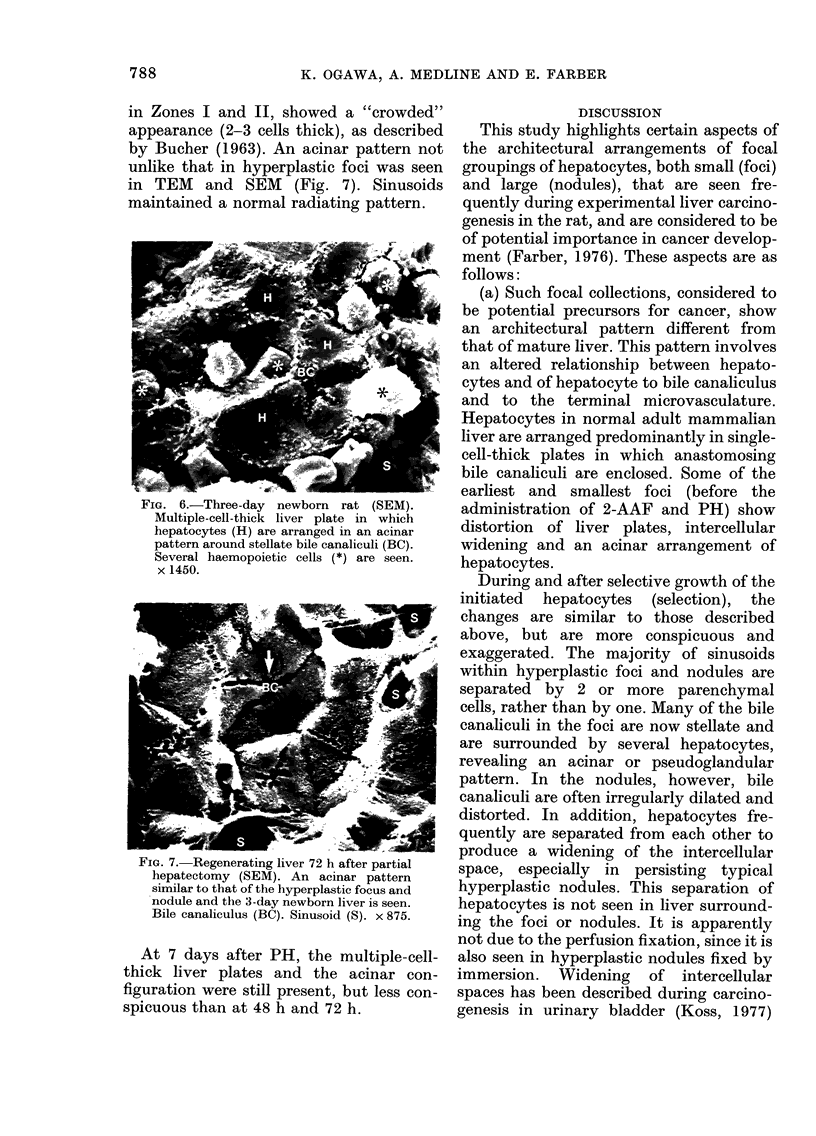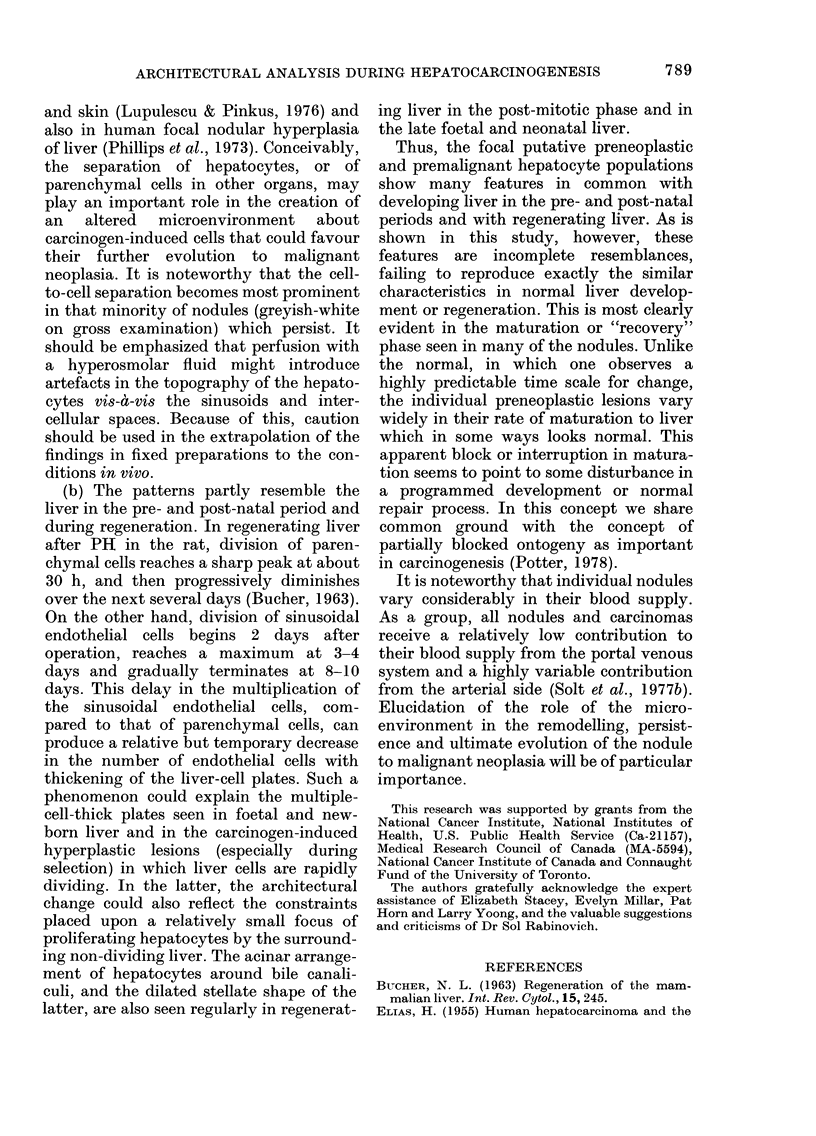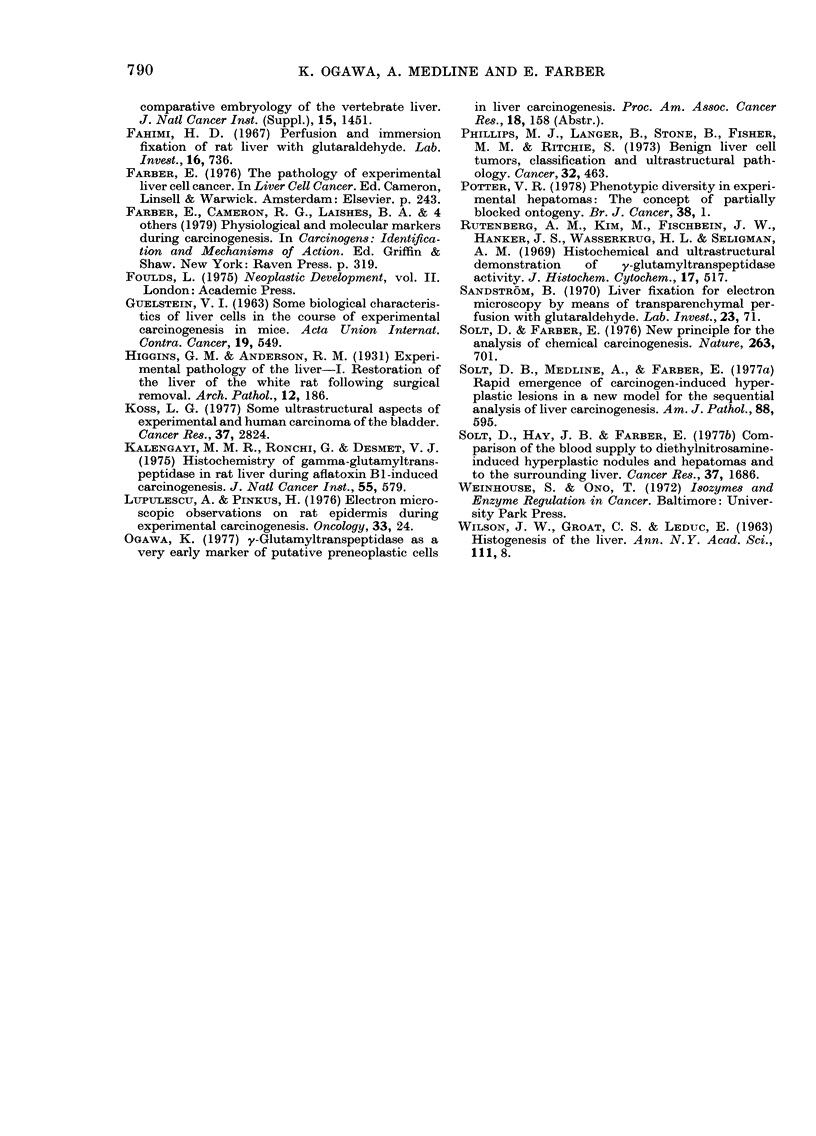# Sequential analysis of hepatic carcinogenesis: the comparative architecture of preneoplastic, malignant, prenatal, postnatal and regenerating liver.

**DOI:** 10.1038/bjc.1979.261

**Published:** 1979-11

**Authors:** K. Ogawa, A. Medline, E. Farber

## Abstract

**Images:**


Br. J. Cancer (1979) 40, 782

### SEQUENTIAL ANALYSIS OF HEPATIC CARCINOGENESIS: THE COMPARATIVE ARCHITECTURE OF PRENEOPLASTIC, MALIGNANT, PRENATAL, POSTNATAL AND REGENERATING LIVER

K. OGAWA*, A. MEDLINE† AND E. FARBER*

*From the *Department of Pathology, Banting Institute, University of Toronto, and the †Department of Pathology, Toronto Western Hospital, Toronto, Ontario, Canada*

Received 14 May 1979 Accepted 20 July 1979

**Summary.—The organizational pattern of hepatocytes in hyperplastic nodules, probable precursors of hepatocellular carcinoma, was examined sequentially at different stages in the carcinogenic process, and compared with the patterns in hepatocellular carcinomas, in developing liver and in regenerating liver. Scanning as well as transmission electron microscopy, and histochemistry with light microscopy were used. The hepatocytes in the hyperplastic lesions were arranged in plates 2 or more cells thick and glands, in contrast to the one-cell-thick plates of hepatocytes in normal mature liver, and showed unusual separation from each other, with irregularly dilated bile canaliculi. The organizational pattern found in the hyperplastic lesions shared properties with developing liver in the perinatal period, regenerating liver following the peak of cell division, and some hepatocellular carcinomas. Unlike the normal, in which there is a highly predictable time scale for change, an apparent delay or interruption in maturation may be of importance in lesions that persist and ultimately evolve into hepatocellular carcinoma.**

THERE IS an increasing realization that many types of human and experimental malignant neoplasms, including hepatocellular carcinoma, are associated with one or more phenotypic characteristics of embryonal, foetal or regenerating cells or tissues (Weinhouse & Ono, 1972). Although these so-called "inappropriate expressions of genetic information" are often considered to be the result of altered gene organization or control intimately associated with malignant behaviour of neoplastic cells, there is little direct evidence to support this view. Since the development of a neoplasm in most tissues and organs, especially epithelial carcinoma, is often a gradual process which appears to involve a series of sequential cellular alterations during preneoplastic and premalignant phases (Foulds, 1975), it is of fundamental importance to determine which altered phenotypic expressions are acquired early and which only late in the process.

In a continuing study involving the sequential analysis of the biology and biochemistry of the different cell populations during chemically induced cancer in the rat, it has become evident that some markers characteristic of embryonal or foetal phases of normal development appear very early in the carcinogenic process, and long before the appearance of overt cancer (Farber *et al.*, 1979). Since contiguous cell-to-cell relationships are phenotypic manifestations of cell and tissue modulation during normal development and in pathological processes, and since haptocellular carcinomas and late hyperplastic nodules are composed of hepatocyte populations with different architectural patterns (Elias, 1955; Farber, 1976) that distinguish them from mature liver, it was of interest and importance to

Correspondence: Dr E. Farber, Department of Pathology, University of Toronto, Toronto, Ontario, Canada, M5G IL5

determine at what step in the carcinogenic process this altered organization is manifested.

Recently a new model of liver carcinogenesis has been developing, which enables an improved control of the early steps considered to be involved in the development of cancer with chemicals (Solt & Farber, 1976; Solt *et al.*, 1977*a*). It is based on the hypothesis that carcinogen-induced presumptive initiated hepatocytes have not acquired any autonomy for growth (Farber, 1976; Farber *et al.*, 1979) but have acquired a resistance to certain cytotoxic effects of carcinogens, in such a way that they are able to proliferate in a carcinogen-induced environment which inhibits the proliferation of normal or unintiated hepatocytes (Guelstein, 1963; Farber, 1976).

Carcinogenesis is initiated by a single dose of carcinogen such as diethylnitrosamine (DEN). The relatively small number of altered hepatocytes so induced is selectively stimulated to proliferate vigorously by partial hepatectomy (PH) coupled with dietary exposure to 2-acetylaminofluorene (2-AAF) for a brief period before and after PH, an environment that inhibits the regenerating response of most hepatocytes (Solt & Farber, 1976). The few altered hepatocytes grow rapidly and synchronously, and within one week after PH become grossly visible small nodules. After this period of rapid growth, which usually is completed by about 2–3 weeks, the majority of nodules undergo a process of remodelling or maturation to normal-appearing liver at variable rates over the next few months. A few nodules persist as such. By 9 months (*i.e.* 8 months after the discontinuation of the brief exposure to 2-AAF) at least 70% of the animals develop one or more hepatocellular carcinomas, sometimes apparently arising within hyperplastic nodules. In comparison with other models of liver carcinogenesis, an additional feature of this model is the relatively normal appearance of the surrounding liver for most of the duration of the process (Solt *et al.*, 1977*a*). It should be emphasized that, with adult rats, the brief exposure to the 2-AAF under the conditions used does not induce any demonstrable resistant hepatocytes and no proliferating foci, nodules or cancer develop in the absence of the initiating dose of DEN (Solt *et al.*, 1977*a*).

In this communication, the structural patterns of hepatocytes in early and late preneoplastic foci and nodules are described, in comparison with the patterns in some hepatocellular carcinomas, in developing foetal and neonatal liver, and in regenerating liver. The possible significance of selected changes to the carcinogenic process is briefly discussed.

### MATERIALS AND METHODS

Male Fischer-344 rats (Charles River) weighing 120–130 g were maintained on a high (24%) protein semi-synthetic basal diet (Bio-Serv Inc.). The animals were exposed to a daily cycle of 12 h light and 12 h darkness, and acclimatized to the environment for at least one week before the onset of the experiments.

To study developing liver, pregnant rats of the Fischer-344 strain were received in early gestation and maintained as above.

*Carcinogenic treatment.* — Hyperplastic lesions were induced by the regimen reported by Solt *et al.* (1977*a*). The rats received DEN i.p. at a dose of 200 mg/kg body wt, and were maintained on the basal diet for 2 weeks. 2-AAF (0·02%) was then added to the basal diet for a further 2 weeks. The animals were subjected to a two-thirds PH (Higgins & Anderson, 1931) after 7 days on 2-AAF (Day 21 of the experiment). Groups of 2–3 rats were killed at 2, 4, 10, 12, 19, 28 and 52 weeks after DEN. The animals were not fasted before being killed, except for the group which were killed 2 weeks after DEN administration. This latter group was fasted in order to detect, with the periodic-acid–Schiff stain (PAS), very early lesions in which individual cells retained their cytoplasmic glycogen after fasting for 24 h, while the surrounding liver lost virtually all its glycogen and became PAS-negative.

*Developing liver.*—Foetal rat livers were obtained from pregnant rats on the 15th, 18th and 21st day of gestation. Newborn rats were killed on Days 1, 3 and 7 after birth.

*Regenerating liver*.—To compare hyperplastic lesions with normal regenerating liver, rats weighing 120–130 g underwent two-thirds PH. Two rats were killed at each interval, *viz*. at 3 and 6 h and at 1, 1·5, 2, 3 and 7 days after PH.

*Transmission electron microscopy* (*TEM*).—All adult livers were fixed by perfusion fixation (Fahimi, 1967). Livers of adult rats were initially perfused through the portal vein with 0·9% NaCl solution, followed by a fixative containing 2% glutaraldehyde and 4% formaldehyde in a 0·1M cacodylate buffer at pH 7·4 (5–6 ml/min). In animals with hepatocellular carcinomas, the liver was perfused with 0·9% NaCl solution *via* the portal vein and subsequently perfused in a retrograde direction *via* the hepatic vein by flushing with 0·9% NaCl solution, followed by the fixative. This was done to improve fixation, since hepatomas are fixed poorly by perfusion through the portal vein, most probably owing to a relative decrease in the portal blood supply (Solt *et al*., 1977*b*).

In developing liver, fixation was performed by transparenchymal perfusion using the method described by Sandström (1970). Dissected pieces of fixed tissue were then placed in the same fixative for 4–6 h, post-fixed in 1% osmium and embedded in Epon and Araldite. Semi-thin sections of plastic-embedded tissue were stained with toluidine blue.

For detection of very early lesions induced by DEN alone, the sections were stained with PAS and toluidine blue. Ultra-thin sections were cut on a Porter MT2 ultramicrotome, stained with uranyl acetate and lead citrate, and examined under a Phillips 301 electron microscope.

*Scanning electron microscopy* (*SEM*).—Scanning electron microscopic studies were performed on normal, newborn and regenerating liver, and on hyperplastic and neoplastic lesions at various times during the carcinogenic process.

Liver was fixed by perfusion fixation as described above. After dehydration with ethanol, small pieces of tissue ~1 × 1 × 3 mm were manually fractured under a dissecting microscope, subjected to critical-point drying, coated with gold–palladium and viewed in a Coates-Welter 101 scanning electron microscope at 10–15 kV.

*Histochemistry*.—The presence of γ-glutamyltranspeptidase (GGT) was used to visualize the organizational pattern of hepatocytes and their relationship to bile canaliculi (Kalengayi *et al*., 1975). Histochemical staining for GGT was performed by the method of Rutenberg *et al*. (1969).

### RESULTS

### *Islands of altered hepatocytes* (*2 weeks after onset of experiment*)

Occasional isolated small aggregates of altered hepatocytes were present in the liver within 2 weeks after DEN administration. The lesions were easily demonstrated histochemically with GGT, but

FIG. 1.—A small island of altered hepatocytes 2 weeks after administration of diethylnitrosamine (DEN). (a): The cells contain PAS+ material (within the confines of the arrows) despite fasting. Liver plates and sinusoids are distorted and intercellular spaces are widened within the focus relative to the surrounding parenchyma (S). Note the acinar arrangement of hepatocytes (curved arrow). PAS–Toluidine blue. × 235. (b): γ-Glutamyltranspeptidase (GGT) activity predominantly staining bile canalicular membranes. × 235.

FIG. 2.—Hyperplastic focus 4 weeks after DEN. (a): Liver plates within a focus (F) are several cells thick and lack a normal radiating pattern. Note dilated bile canaliculi (arrows). Normal surrounding liver (S) at extreme right. Toluidine blue. × 230. (b): GGT staining showing a stellate bile canalicular pattern. × 230. (c): Scanning electron micrograph (SEM) showing multiple-cell-thick liver plates with an acinar arrangement of hepatocytes around a central stellate canaliculus (BC). Note the sinusoids (S) separated by several liver cells. × 1380. (d): SEM of fractured surface of normal liver. Sinusoids (S) are seen on both sides of the one-cell-thick liver plate. Note the hemi-bile canaliculus (BC) traversing the central area of the liver cells. Red blood cell (RBC). Kupffer cell (K). × 1530.

exceedingly difficult to visualize in haematoxylin and eosin sections. About 30% of GGT+ lesions showed abundant PAS+ cytoplasmic material after 24 h of starvation, which was a useful marker for detection of these tiny foci. An organizational change of liver cells was not detectable in the very small lesions consisting of only a few hepatocytes. However, in the lesions 10 or more hepatocytes in diameter, as seen in Fig. 1(a), hepatic plates were 2–3 cells thick and an acinar pattern of the hepatocytes was seen. The hepatic plates and sinusoids were distorted. The intercellular spaces were widened to varying degrees. The pattern of GGT staining was predominantly canalicular (Fig. 1(b)).

*Hyperplastic foci* (4 *weeks after onset of experiment*)

One week after PH, the lesions described above showed preferential growth and became visible on gross inspection, measuring up to 1 mm in diameter. The hyperplastic foci were generally spherical and translucent, compressing the surrounding normal hepatic parenchyma. Liver cells were characteristically arranged in 2–3-cell-thick plates and showed complete loss of a radiating pattern, compared to normal or surrounding liver (Fig. 2(a)).

As seen in Fig. 2(b), the bile canaliculi showed stellate patterns with prominent GGT activity.

An acinar or pseudoglandular arrangement of hepatocytes around the dilated stellate bile canaliculi was a prominent feature with TEM and SEM (Fig. 2(c)), in comparison with normal liver (Fig. 2(d)).

The sinusoids were variable in size and infrequently dilated, with loss of the normal radiating pattern.

*Hyperplastic nodules* (10, 12, 19, 28 *and* 52 *weeks after onset of experiment*)

The liver contained significant numbers of greyish-white typical hyperplastic nodules, 2–5 mm in diameter. The nodules were composed of hepatocytes arranged predominantly in 2–3-cell-thick plates. The nodules, as seen in Fig. 3, commonly showed widening of intercellular spaces. The canaliculi showed strong GGT activity and assumed an irregular shape, compared to the stellate configuration seen in hyperplastic foci. SEM revealed an irregular arrangement of hepatocytes and variably shaped dilated bile canaliculi on the fractured cell surfaces.

The sinusoids within the nodule were dilated and distorted. At the junction of the nodule and the surrounding parenchyma, occasional bile ductules and blood vessels were seen. Also, a few small thin-walled blood vessels were seen within the nodules.

Many of the lesions showed histochemical and architectural features of remodelling or maturation (Ogawa, 1977). The remodelling lesions were characterized by patchy disappearance of GGT staining (Fig. 4). GGT staining revealed a branching pattern rather than the irregular acinar arrangement seen in typical hyperplastic nodules. Hepatocytes were arranged predominantly in the form of single-cell-thick plates, and only minimal and focal intercellular widening was apparent. The hepatocytes in the remodelling nodules frequently showed a marked increase in

FIG. 3.—TEM of hyperplastic nodule 10 weeks after DEN. The hepatocytes are irregular in shape, with a variable degree of intercellular separation (arrows). The bile canaliculus (BC) is widened and surrounded by an acinar arrangement of hepatocytes. Sinusoids (S). × 900.

Fig. 4.—Hyperplastic nodule 10 weeks after DEN showing features of remodelling, with patchy disappearance of GGT staining. Bile canalicular activity reveals a branching linear pattern (arrows). × 235.

Fig. 5.—SEM of trabecular form of hepatocellular carcinoma 52 weeks after DEN. Blood vessels (BV) are separated by several neoplastic cells. × 600.

the amount of cytoplasmic glycogen and/or lipid, causing narrowing of the sinusoids.

*Hepatocellular carcinoma*

The organizational pattern of hepatocellular carcinoma was much more variable than the hyperplastic foci and typical nodules. A trabecular pattern predominated with hepatoma cells in multiple-cell-thick plates or cords of up to 10 cells, separated by irregular distended sinusoids (Fig. 5).

An acinar pattern was found within many of the cancers, in which the hepatocytes were arranged either around stellate canaliculi, as in hyperplastic foci, or around dilated and irregular canaliculi, as in hyperplastic nodules. Cell separation, which was so striking a feature in the hyperplastic nodules, was not prominent.

GGT activity was not uniformly demonstrated, but where visualized was confined to canaliculi, cell membranes and cytoplasm.

*Developing liver*

Observations of the sequence of changes in the developing rat liver were essentially those described in other studies (Wilson *et al.*, 1963). On light microscopy at 15 days of gestation, an organizational pattern of hepatocytes could not be recognized because of the presence of numerous haemopoietic cells.

Low-magnification electron microscopy revealed irregularly shaped hepatocytes joined by elongated cytoplasmic processes. There was considerable compression of the liver cells by many adjacent haemopoietic cells. Only a small number of sinusoids were seen, and these were dilated and contained numerous haemopoietic cells.

With maturation up to the 21st day of gestation, the hepatocytes assumed a tubular arrangement, with liver cells surrounding dilated bile canaliculi. The latter showed strong GGT activity, displaying both a branching and stellate pattern. At this time, haemopoietic cells decreased in number and localized predominantly on the sinusoidal aspect of liver plates in the space of Disse.

After birth, the 2–3-cell-thick plates associated with an acinar configuration were maintained (Fig. 6), but were less obvious by one week.

*Regenerating liver*

Three to 24 h after PH, the organizational pattern could not be distinguished from that of normal liver, apart from slight widening of the intercellular spaces, seen clearly at 3 and 6 h, but no longer evident at 24 h.

At 48 and 72 h, liver plates, especially in Zones I and II, showed a "crowded" appearance (2–3 cells thick), as described by Bucher (1963). An acinar pattern not unlike that in hyperplastic foci was seen in TEM and SEM (Fig. 7). Sinusoids maintained a normal radiating pattern.

FIG. 6.—Three-day newborn rat (SEM). Multiple-cell-thick liver plate in which hepatocytes (H) are arranged in an acinar pattern around stellate bile canaliculi (BC). Several haemopoietic cells (*) are seen. × 1450.

FIG. 7.—Regenerating liver 72 h after partial hepatectomy (SEM). An acinar pattern similar to that of the hyperplastic focus and nodule and the 3-day newborn liver is seen. Bile canaliculus (BC). Sinusoid (S). × 875.

At 7 days after PH, the multiple-cell-thick liver plates and the acinar configuration were still present, but less conspicuous than at 48 h and 72 h.

### DISCUSSION

This study highlights certain aspects of the architectural arrangements of focal groupings of hepatocytes, both small (foci) and large (nodules), that are seen frequently during experimental liver carcinogenesis in the rat, and are considered to be of potential importance in cancer development (Farber, 1976). These aspects are as follows:

(a) Such focal collections, considered to be potential precursors for cancer, show an architectural pattern different from that of mature liver. This pattern involves an altered relationship between hepatocytes and of hepatocyte to bile canaliculus and to the terminal microvasculature. Hepatocytes in normal adult mammalian liver are arranged predominantly in single-cell-thick plates in which anastomosing bile canaliculi are enclosed. Some of the earliest and smallest foci (before the administration of 2-AAF and PH) show distortion of liver plates, intercellular widening and an acinar arrangement of hepatocytes.

During and after selective growth of the initiated hepatocytes (selection), the changes are similar to those described above, but are more conspicuous and exaggerated. The majority of sinusoids within hyperplastic foci and nodules are separated by 2 or more parenchymal cells, rather than by one. Many of the bile canaliculi in the foci are now stellate and are surrounded by several hepatocytes, revealing an acinar or pseudoglandular pattern. In the nodules, however, bile canaliculi are often irregularly dilated and distorted. In addition, hepatocytes frequently are separated from each other to produce a widening of the intercellular space, especially in persisting typical hyperplastic nodules. This separation of hepatocytes is not seen in liver surrounding the foci or nodules. It is apparently not due to the perfusion fixation, since it is also seen in hyperplastic nodules fixed by immersion. Widening of intercellular spaces has been described during carcinogenesis in urinary bladder (Koss, 1977)

and skin (Lupulescu & Pinkus, 1976) and also in human focal nodular hyperplasia of liver (Phillips *et al.*, 1973). Conceivably, the separation of hepatocytes, or of parenchymal cells in other organs, may play an important role in the creation of an altered microenvironment about carcinogen-induced cells that could favour their further evolution to malignant neoplasia. It is noteworthy that the cell-to-cell separation becomes most prominent in that minority of nodules (greyish-white on gross examination) which persist. It should be emphasized that perfusion with a hyperosmolar fluid might introduce artefacts in the topography of the hepatocytes *vis-à-vis* the sinusoids and intercellular spaces. Because of this, caution should be used in the extrapolation of the findings in fixed preparations to the conditions *in vivo*.

(b) The patterns partly resemble the liver in the pre- and post-natal period and during regeneration. In regenerating liver after PH in the rat, division of parenchymal cells reaches a sharp peak at about 30 h, and then progressively diminishes over the next several days (Bucher, 1963). On the other hand, division of sinusoidal endothelial cells begins 2 days after operation, reaches a maximum at 3–4 days and gradually terminates at 8–10 days. This delay in the multiplication of the sinusoidal endothelial cells, compared to that of parenchymal cells, can produce a relative but temporary decrease in the number of endothelial cells with thickening of the liver-cell plates. Such a phenomenon could explain the multiple-cell-thick plates seen in foetal and newborn liver and in the carcinogen-induced hyperplastic lesions (especially during selection) in which liver cells are rapidly dividing. In the latter, the architectural change could also reflect the constraints placed upon a relatively small focus of proliferating hepatocytes by the surrounding non-dividing liver. The acinar arrangement of hepatocytes around bile canaliculi, and the dilated stellate shape of the latter, are also seen regularly in regenerating liver in the post-mitotic phase and in the late foetal and neonatal liver.

Thus, the focal putative preneoplastic and premalignant hepatocyte populations show many features in common with developing liver in the pre- and post-natal periods and with regenerating liver. As is shown in this study, however, these features are incomplete resemblances, failing to reproduce exactly the similar characteristics in normal liver development or regeneration. This is most clearly evident in the maturation or "recovery" phase seen in many of the nodules. Unlike the normal, in which one observes a highly predictable time scale for change, the individual preneoplastic lesions vary widely in their rate of maturation to liver which in some ways looks normal. This apparent block or interruption in maturation seems to point to some disturbance in a programmed development or normal repair process. In this concept we share common ground with the concept of partially blocked ontogeny as important in carcinogenesis (Potter, 1978).

It is noteworthy that individual nodules vary considerably in their blood supply. As a group, all nodules and carcinomas receive a relatively low contribution to their blood supply from the portal venous system and a highly variable contribution from the arterial side (Solt *et al.*, 1977*b*). Elucidation of the role of the microenvironment in the remodelling, persistence and ultimate evolution of the nodule to malignant neoplasia will be of particular importance.

This research was supported by grants from the National Cancer Institute, National Institutes of Health, U.S. Public Health Service (Ca-21157), Medical Research Council of Canada (MA-5594), National Cancer Institute of Canada and Connaught Fund of the University of Toronto.

The authors gratefully acknowledge the expert assistance of Elizabeth Stacey, Evelyn Millar, Pat Horn and Larry Yoong, and the valuable suggestions and criticisms of Dr Sol Rabinovich.

### REFERENCES

BUCHER, N. L. (1963) Regeneration of the mammalian liver. *Int. Rev. Cytol.*, **15**, 245.

ELIAS, H. (1955) Human hepatocarcinoma and the comparative embryology of the vertebrate liver. *J. Natl Cancer Inst.* (Suppl.), **15**, 1451.

FAHIMI, H. D. (1967) Perfusion and immersion fixation of rat liver with glutaraldehyde. *Lab. Invest.*, **16**, 736.

FARBER, E. (1976) The pathology of experimental liver cell cancer. In *Liver Cell Cancer*. Ed. Cameron, Linsell & Warwick. Amsterdam: Elsevier. p. 243.

FARBER, E., CAMERON, R. G., LAISHES, B. A. & 4 others (1979) Physiological and molecular markers during carcinogenesis. In *Carcinogens: Identification and Mechanisms of Action*. Ed. Griffin & Shaw. New York: Raven Press. p. 319.

FOULDS, L. (1975) *Neoplastic Development*, vol. II. London: Academic Press.

GUELSTEIN, V. I. (1963) Some biological characteristics of liver cells in the course of experimental carcinogenesis in mice. *Acta Union Internat. Contra. Cancer*, **19**, 549.

HIGGINS, G. M. & ANDERSON, R. M. (1931) Experimental pathology of the liver—I. Restoration of the liver of the white rat following surgical removal. *Arch. Pathol.*, **12**, 186.

KOSS, L. G. (1977) Some ultrastructural aspects of experimental and human carcinoma of the bladder. *Cancer Res.*, **37**, 2824.

KALENGAYI, M. M. R., RONCHI, G. & DESMET, V. J. (1975) Histochemistry of gamma-glutamyltranspeptidase in rat liver during aflatoxin B1-induced carcinogenesis. *J. Natl Cancer Inst.*, **55**, 579.

LUPULESCU, A. & PINKUS, H. (1976) Electron microscopic observations on rat epidermis during experimental carcinogenesis. *Oncology*, **33**, 24.

OGAWA, K. (1977) $\gamma$-Glutamyltranspeptidase as a very early marker of putative preneoplastic cells in liver carcinogenesis. *Proc. Am. Assoc. Cancer Res.*, **18**, 158 (Abstr.).

PHILLIPS, M. J., LANGER, B., STONE, B., FISHER, M. M. & RITCHIE, S. (1973) Benign liver cell tumors, classification and ultrastructural pathology. *Cancer*, **32**, 463.

POTTER, V. R. (1978) Phenotypic diversity in experimental hepatomas: The concept of partially blocked ontogeny. *Br. J. Cancer*, **38**, 1.

RUTENBERG, A. M., KIM, M., FISCHBEIN, J. W., HANKER, J. S., WASSERKRUG, H. L. & SELIGMAN, A. M. (1969) Histochemical and ultrastructural demonstration of $\gamma$-glutamyltranspeptidase activity. *J. Histochem. Cytochem.*, **17**, 517.

SANDSTRÖM, B. (1970) Liver fixation for electron microscopy by means of transparenchymal perfusion with glutaraldehyde. *Lab. Invest.*, **23**, 71.

SOLT, D. & FARBER, E. (1976) New principle for the analysis of chemical carcinogenesis. *Nature*, **263**, 701.

SOLT, D. B., MEDLINE, A., & FARBER, E. (1977*a*) Rapid emergence of carcinogen-induced hyperplastic lesions in a new model for the sequential analysis of liver carcinogenesis. *Am. J. Pathol.*, **88**, 595.

SOLT, D., HAY, J. B. & FARBER, E. (1977*b*) Comparison of the blood supply to diethylnitrosamine-induced hyperplastic nodules and hepatomas and to the surrounding liver. *Cancer Res.*, **37**, 1686.

WEINHOUSE, S. & ONO, T. (1972) *Isozymes and Enzyme Regulation in Cancer*. Baltimore: University Park Press.

WILSON, J. W., GROAT, C. S. & LEDUC, E. (1963) Histogenesis of the liver. *Ann. N.Y. Acad. Sci.*, **111**, 8.